# History of Typhoid Fever, as It Prevailed near Geneva, N. Y., in the Fall and Winter of 1846-47

**Published:** 1847-06

**Authors:** Geo. C. Hay

**Affiliations:** Geneva


*History of Typhoid Fever, as it prevailed near Geneva, N. Y., in the Fall and Winter of* 1846-47. By Geo. C. Hay, M. D., of Geneva.—According to your request, I proceed to give you a brief account of the Typhoid fever, which recently prevailed in this region of country, and especially on the opposite shore of the Seneca lake. In that neighbourhood, fevers of a remitting type had prevailed quite extensively during the latter part of the last summer, and during nearly all the fall months, but not a single case, as I can ascertain, died of it at that time. In December, the first cases of the low form of fever showed themselves. All the cases which have occurred seem to be confined to a neighbourhood of from one to two miles square; and beyond the limits of this, as far as I can learn, no cases have appeared. The symptoms in the forming stage differ but little from an attack of ordinary fever, commencing with loss of appetite, sense of lassitude and disinclination to exercise of any kind, chilliness, pains in bones, etc. After twenty-four or forty-eight hours, headache came on invariably in all the cases, and in many it was the first symptom, and continued very obstinate through the whole course of the disease. About the third day the patients were generally obliged to take to their beds, the headache increasing, attended with vertigo, dimness of vision, ringing in the ears, suffusion of the conjunctiva and deafness. The tongue was at first covered with a thick white fur which changed by the fourth or fifth day into a brown, and finally into a black during the latter stages of the disease. In a great majority of the cases diarrhœa was a prominent symptom from the commencement, so much so that even the use of the mildest febrifuge medicine was inadmissible; this was controlled with great difficulty by opiates, and in some cases astringents had to be resorted to. Accompanying this diarrhœa, we found considerable tenderness of the abdomen in nearly all the cases. The discharges from the bowels were generally very watery, of a dark colour, very fetid, the urine scanty, sometimes entirely suppressed, and of a very red color. The skin was generally quite dry, although not very hot at any time, and in no case could I discover any of the "calor mordax" spoken of by writers, and which I have frequently felt in similar cases. As the disease progressed, in many cases delirium was a constant attendant through the whole course of the fever, and none were entirely free from it. In many cases the collapse came on very suddenly, and in others a gradual sinking came on, and steadily progressed until the patients died. The collapse seemed to bear no relation whatever to the severity of the first stage, as is generally the case, as sometimes in those who had been attacked but slightly the collapse was sudden and fatal, while in those whose stage of excitement had been very severe the collapse came on very gradually, and progressed slowly, and *vice versa*. As to the post-mortem appearance I can give you but little information, as owing to the prejudice existing among the people it was next to impossible to procure an examination. After I had left, however, one examination was made of a boy aged fourteen, who died very suddenly, and I understood from a physician who was present, that his bowels were a complete mass of mortification. As to the treatment, it was very various. All the patients, however, had in the commencement mercury, in some of its forms, and in two cases which have since recovered, it was carried to the extent of slight salivation. Some cases were bled generally, and some not, but it seemed to make but little difference in the continuance or violence of the disease. The local treatment consisted in cold applications, leeches, and blisters to the head and nape of the neck ; cups and blisters to the chest, when the symptoms seemed to demand ; hot fomentations, poultices, leeches, and blisters to the abdomen, etc. In some cases we ordered the patients to be washed over the whole body with a solution of nitro-muriatic acid made as strong as they could bear it ; this was done twice in twenty-four hours, and seemed at least to give considerable temporary relief to the patients. The general treatment was at first calomel, hyd. cum. cretâ, or pil. hyd., followed by the liquor ammoniæ acetatis, or spiritus ætheris nitrici, with the potassæ nitras or vinum ipecacuanhæ or antimonii ; but in some cases, and indeed in many, their use was impossible, on account of the tendency to diarrhœa, as everything taken into the stomach ran off by the bowels in a short time. In all the cases stimulants and tonics had to be resorted to sooner or later, but generally with little benefit, although at first the patients seemed to rally under their influence, yet they seemed to have no permanent effect in many cases. Those used were the infusum serpentariæ, or columbæ, camphor, ammonia, etc., together with sulphate of quinine, port wine and brandy, with a nourishing diet of beef-tea, chicken-jelly, arrowroot, etc. I forgot to mention that the pulse was in most cases very frequent, from the commencement, ranging from 100 to 130 and 140 during the whole time. In some cases the pulse was quite full and hard in the commencement, but generally it was quite small and very easily compressed. I may say in conclusion that it seemed to matter little what course of treatment was pursued, the patients in a large majority of cases died, some during the first week, others running on four, six, eight, and ten weeks. In one family, five persons died, all between the ages of fourteen and thirty ; indeed nearly all the cases have been young persons.—*New York Jour. of Med.*

